# Dose and time-dependent sub-chronic toxicity study of hydroethanolic leaf extract of *Flabellaria paniculata* Cav. (Malpighiaceae) in rodents

**DOI:** 10.3389/fphar.2014.00078

**Published:** 2014-04-23

**Authors:** Abidemi J. Akindele, Adejuwon A. Adeneye, Oluwole S. Salau, Margaret O. Sofidiya, Adokiye S. Benebo

**Affiliations:** ^1^Department of Pharmacology, Therapeutics and Toxicology, Faculty of Basic Medical Sciences, College of Medicine, University of LagosLagos, Nigeria; ^2^Department of Pharmacology, Faculty of Basic Medical Sciences, Lagos State University College of MedicineLagos, Nigeria; ^3^Department of Pharmacognosy, Faculty of Pharmacy, University of LagosLagos, Nigeria; ^4^Department of Pathology and Forensic Medicine, Faculty of Basic Medical Sciences, Lagos State University College of MedicineLagos, Nigeria

**Keywords:** *Flabellaria paniculata*, Malpighiaceae, acute toxicity, sub-chronic toxicity, reversibility study

## Abstract

*Flabellaria paniculata* Cav. (Malpighiaceae) is a climbing shrub, the preparations of which are used in the treatment of wounds and ulcers in Nigeria and Ghana. This study investigated the sub-chronic toxicity profile of the hydroethanolic leaf extract of *F. paniculata* (HLE-FP). HLE-FP was administered *p.o*. (20, 100, and 500 mg/kg) for 30 and 60 days to different groups of rats. Control animals received 10 ml/kg distilled water. In the group of animals for reversibility study, HLE-FP administration ceased on the 60th day and animals were monitored for a further 15 days. Results showed that oral treatment with HLE-FP for 30 days caused significant (*p* < 0.05) reductions in weight gain pattern compared to control. These changes were sustained with 60 days treatment. However, no significant (*p* > 0.05) differences in relative organ weights between control and treatment groups were observed. HLE-FP-treated rats showed significant (*p* < 0.05) increases in Hb, PCV and RBC on day 30 and significant (*p* < 0.05) increases in MCV and MCH indices on day 60 compared to control. There were significant (*p* < 0.05) elevations in serum K^+^, urea and creatinine compared to control. The liver function tests showed slight but non-significant alterations in relevant parameters when compared to control. Biochemical findings were supported by histopathological observations of vital organs including the kidney and liver. Toxicities observed in respect of kidney function were irreversible at 15 days of stoppage of treatment. In the acute toxicity study, HLE-FP given *p.o*. caused no lethality at 5000 mg/kg but behavioral manifestations like restlessness, generalized body tremor, feed, and water refusal were observed. The *i.p*. LD_50_ was estimated to be 2951.2 mg/kg. Findings in this study showed that HLE-FP is relatively non-toxic on acute exposure and generally safe on sub-chronic administration, but could be deleterious on the kidneys on prolonged oral exposure at a high dose. Thus, caution should be exercised with its long-term usage.

## Introduction

Mankind has relied on plants for food and medicines as early as his emergence and this continues to be the case in this modern times. By WHO estimation, about 80% of the population in developing countries depend on traditional medicine, most significantly herbal remedies, for their primary healthcare (World Health Organization, 2003; Campbell-Tofte et al., 2012). This is very true for significant populations in Africa, Asia, and South America especially. According to Leonti and Casu (2013), traditional medicine and medicinal plants are frequently used in urban settings as alternatives in daily health care and for self-medication against minor and chronic ailments, but especially relied upon in less wealthy rural areas or in times of economic crises. In recent times however, the faith in and patronage of herbal remedies has tremendously increased even in the developed countries, as observed in Europe and North America. According to Campbell-Tofte et al. (2012), plant-derived traditional medicines have become very lucrative business in the international marketplace and annual revenues reported for the sale of herbal medicines in Western Europe reached US$5 billion in 2003–2004.

A major reason for the increasing patronage of herbal remedies across the world, aside from belief of efficacy or proven efficacy, is general perception of safety. A significant number of the general public across the world hold the false perception that traditional medicinal products are safer because they are prepared from natural sources without much processing (Calixto, 2000) and due to history of long usage in the treatment of diseases according to knowledge accumulated over centuries (Fennell et al., 2004). However, it has been reported that many plants used as food or in traditional medicine are potentially toxic, mutagenic and carcinogenic based on reports of scientific research (Kassie et al., 1996; De Sã Ferrira and Ferrão Vargas, 1999; Fennell et al., 2004). Adewunmi and Ojewole (2004) also reported that some traditional, complementary, and alternative medicines contain toxic and potentially lethal constituents which include aristolochic acids, pyrrolizidine alkaloids, benzophenanthrine alkaloids, lectins, viscotoxins, saponins, diterpenes, cyanogenic glycosides and furanocoumarins.

According to Campbell-Tofte et al. (2012), WHO has called for urgency in setting up international standards and methods for the evaluation of safety and therapeutic efficacy of traditional medicinal approaches, in recognition of the potential of traditional medicine for providing treatment solutions for various infectious and chronic diseases. Despite the fact that pharmacological and toxicological evaluations of medicinal plants are essential for drug/standardized herbal remedy development (Ibarrola et al., 2000; Ahmed et al., 2006; Perera et al., 2010; Afolabi et al., 2012), the reports of scientific investigations detailing efficacy based on traditional claims far outnumber those of toxicological investigations and toxicity. Verpoorte et al. (2005) and Campbell-Tofte et al. (2012) have suggested that plant preparations and phytochemicals derived from medicinal plants pre-selected in traditional medicine be investigated *in vivo* for their safety and efficacy using carefully selected disease model animals, or in specially-designed randomized and placebo-controlled clinical trials. It is unarguable that safety evaluations of herbal remedies, especially to determine the consequence of prolonged use, are critical to the discovery and development of standardized herbal remedies and the acceptance of such by stakeholders in healthcare for therapeutic application.

*Flabellaria paniculata* Cav. (Malpighiaceae) is a climbing shrub, with gray-silky branchlets/leaves and white to light pink flowers, widely distributed in equatorial Africa (from Senegal to W Cameroons and Equatorial Guinea; and across the Congo basin to Uganda and Tanzania) in thickets, woodlands, and forests, especially along rivers (Hutchinson and Dalziel, 1958; Burkill, 1997). The plant is locally called “Tinupogbe” and “Ajidere” by the Yoruba people of Southwest Nigeria. In terms of uses in traditional African medicine (TAM), preparations from different parts of the plant are used for general healing (leaf), as abortifacients, ecbolics; for menstrual cycle disorder (leaf-sap); and as antidotes for venomous stings, bites etc. (root) (Burkill, 1997). According to Burkill (1997), the leaf is used in Ghana and Nigeria on wounds and sores; in Ivory Coast the leaf-sap is frequently taken for amenorrhea and as an ecbolic; the ground root along with some other ingredients is used as a vaccine for snake-bite in the Mampong area of Ghana. Based on the report of Sofidiya et al. (2012), the leaves and root of *F. paniculata* are used as ingredients of herbal preparations for the treatment of wound and ulcers sold and prescribed in herbal stalls in Lagos metropolis of Nigeria.

In terms of scientific evaluation, Abo and Olugbuyiro (2004a,b) and Olugbuyiro et al. (2010) reported the wound healing and antibacterial activities of the leaf extracts of the plant. Based on reported traditional use, Sofidiya et al. (2012) investigated the effectiveness of the ethanolic leaf and root extracts of *F. paniculata* in rat models of gastric ulcer. The authors concluded that the ethanolic leaf extract and not the ethanolic root extract possess antiulcer activity. Both extracts were found to contain terpenoids, tannins and saponins but not alkaloids. Flavonoids were reported to be additionally present in the leaf extract while anthraquinones were detected in the root extract suggesting that the antiulcer effect of the leaf extract may be associated with the presence of flavonoids which are known to possess antioxidant property.

In view of the wide application of leaf preparations of *F. paniculata* in TAM and tendency for overuse and prolong intake, this study was designed to investigate the possible dose and time-dependent sub-chronic toxicity effects of the hydroethanolic leaf extract of *F. paniculata* (HLE-FP) in rodents.

## Materials and methods

### Plant collection

Fresh leaves of *F. paniculata* were collected from a deciduous forest in Ilaro Town, Egbado Local Government Area of Ogun State, Nigeria. The plant samples collected in July 2012 were identified and authenticated by Mr. TK Odewo, a former Senior Superintendent of the Forestry Research Institute of Nigeria (FRIN), Ibadan, Nigeria, now Herbarium Curator in the Department of Botany and Microbiology, Faculty of Science, University of Lagos, Lagos, Nigeria. A voucher specimen numbered LUH 5009 was deposited in the institutional herbarium for reference purposes.

### Plant extraction

The fresh leaves of *F. paniculata* were air-dried at room temperature (23–25°C) until a constant weight was consistently obtained. The dried materials were ground into coarse powder using Christy and Norris 8' Laboratory Milling Machine (serial no. 50158). One hundred gram of the dried leaf material was macerated in 1 L of hydroethanol (1:1) with continuous mechanical stirring for 3 h after which the extract was decanted and filtered using Whatman No. 1 paper. The process was repeated with the residue and subsequent residue (×2) to ensure exhaustive extraction. The combined filtrate was evaporated to dryness under vacuum on a rotary evaporator (Heidolph-Rotacool, Germany) at 40°C giving a deep brown, sweet-smelling solid residue. The extract was reconstituted in normal saline before administration to experimental animals.

### Preliminary phytochemical analysis

This was carried out for the detection of various phytoconstituents according to the established method of Sofowora (1993).

### Experimental animals

The animals used in this study, including young adult female Wistar albino rats (6–8 weeks old; 80–125 g) and Swiss albino mice (8–10 weeks old; 20–25 g) were obtained from the Laboratory Animal Centre of the College of Medicine, University of Lagos, Lagos, Nigeria.

The animals were housed in adequately ventilated hygienic compartments, sustained under standard environmental conditions (23–25°C, 12 h/12 h light/dark cycle), and fed with standard rodent diet (Livestock Feeds PLC, Ikeja, Lagos, Nigeria) and water *ad libitum*. The cage beddings and water bottles were cleaned on a daily basis. The animals were allowed 2 weeks of acclimatization before the commencement of experimental procedures.

The investigational procedures adopted in this study were in accordance with the requirements of the Experimentation Ethics Committee on Animal Use of the College of Medicine, University of Lagos, Lagos, Nigeria and the United States National Academy of Sciences Guide for the Care and Use of Laboratory Animals [National Research Council (US) Committee for the Update of the Guide for the Care and Use of Laboratory Animals, 2011].

### Acute toxicity studies

Acute oral (*p.o.*) toxicity study was carried out according to the procedure of the limit dose test of Up-and-Down method (OECD, 2008b) using a computer-guided statistical programme (AOT425statPgm; version 1.0) at a limit dose of 5000 mg/kg body weight/oral route and default of Sigma at 0.5 (Adeneye et al., 2006). Three female rats selected out of a population of 10 rats using systematic randomization techniques, such that the weight differences do not exceed ±10% of the mean initial weight of the sample population, were used in this study. The animals were observed at different time intervals for behavioral manifestations.

Acute intraperitoneal (*i.p.*) toxicity study was conducted using the log dose-probit analysis method of Miller and Tainter (Randhawa, 2009). Mice were randomly allotted to 6 groups of 5 mice each such that the weight difference within and between groups does not exceed ±20% of the sample population. Mice in the different groups were separately treated *i.p*. with normal saline (10 ml/kg) and HLE-FP at doses of 250, 500, 1000, 2000, and 4000 mg/kg. Animals were observed for 2 h post-treatment for behavioral manifestations and mortality within 24 h post-treatment was recorded for the respective groups. The median lethal dose (LD_50_) was estimated using log dose-probit analysis.

### Sub-chronic oral toxicity study

Sixty young adult female Wistar albino rats were randomly divided into 4 major groups (I-IV) with each group sub-divided into 3 sub-groups (A, B, and C) consisting of five rats each. Treatment was then carried out in the different sub-groups as outlined below:

I_A_ and I_B_ (high dose group): 500 mg/kg/day *p.o*. HLE-FP for 30 and 60 days respectively.

II_A_ and II_B_ (medium dose group): 100 mg/kg/day *p.o*. HLE-FP for 30 and 60 days respectively.

III_A_ and III_B_ (low dose group): 20 mg/kg/day *p.o*. HLE-FP for 30 and 60 days respectively.

IV_A_ and IV_B_ (control group): Normal saline 10 ml/kg/day *p.o*. for 30 and 60 days respectively.

I_C_, II_C_, III_C_, and IV_C_ (reversibility study): Normal saline 10 ml/kg/day *p.o*. for 14 days after termination of extract treatments on the 60th day.

### Blood sample collection

A day prior to termination of each set of rats on days 31, 61, and 75 of the experiment, the rats were fasted of feed but drinking water was made available *ad libitum*. The rats were sacrificed humanely under inhaled diethyl ether anaesthesia. After a deep anaesthesia, each rat was mounted on dissecting board and a deep longitudinal incision was made into the ventral surface of the abdomen and thorax. By blunt dissection of the muscles and fasciae, the contents of both the thorax and abdomen were exposed. Blood sample was collected by cardiac puncture with a 21 G needle mounted on a 5 ml syringe plunger. Blood sample for full blood count was collected into EDTA-treated sample bottle. Blood sample for serum biochemical assays was collected into plain sample bottles, allowed 2 h to clot, and centrifuged (Uniscope Laboratory Centrifuge; Model SM 902B, Surgifriend Medicals, England, UK) at 3500 rpm for 10 min to obtain the serum. The serum collected was used to analyse parameters such as alanine aminotransferase (ALT), aspartate aminotransferase (AST), alkaline phosphatase (ALP), total protein (TP), albumin (ALB), total cholesterol (TC), triglycerides (TG), high density lipoprotein cholesterol (HDL-c), electrolytes (sodium, potassium, calcium, bicarbonate and phosphates), urea and creatinine.

### Measurement of body and organ weights

The weight of all rats were taken using electronic Mettler weighing balance (Mettler Toledo Type BD6000, Mettler-Toledo GmbH, Greifensee, Switzerland) on days 1, 15, 30, 45, 60, and 75.

For each animal, the % weight change was calculated as given below:

% Weight change = (Difference between interval and initial body weight ÷ Initial body weight) × 100

The mean of values for each group was then calculated to give % weight change ± *SD*. At the end of the respective treatment periods, the rats were dissected (cut open from the abdominal cavity to the thoracic cavity) under inhaled deep diethyl ether anaesthesia and organs such as the lungs, spleen, stomach, heart, liver, and kidneys were identified, cleared of adherent tissues and harvested. The organs were then rinsed with normal saline, blotted with filter paper and weighed with electronic Mettler weighing balance. The weight of each organ relative to 100 g of the body weight of the rats from which the organs were harvested was calculated as: [organ weight (g) ÷ body weight of rat (g)] × 100.

### Determination of serum biochemical parameters

ALT, AST, ALP, lipids (triglycerides, total cholesterol, HDL-c), total protein, albumin, urea and creatinine were analyzed using standard diagnostic test kits (Randox Laboratories, Crumlin, UK) on Automated Clinical System (Sychron Clinical System®, model: CX5 PRO; Beckman Coulter Inc., Galway, Ireland). Electrolytes (sodium, potassium, chloride, calcium, bicarbonate, and phosphates) were determined using the ISE 6000 BYY SFRI spectrophotometer.

### Hematological assays

Full blood count (FBC) was determined using Hematology Auto-analyzer System (Sysmex Hematology-Coagulation Systems®, Model KX-21N, Sysmex Incorporation, Kobe, Japan).

### Histopathological assessment

Selected organs were identified and carefully extracted. The tissues were fixed in 10% buffered formalin, dehydrated in graded alcohol (70, 90, 95, and 100%) and embedded in paraffin. The embedded tissues were then cut into 4–5 μm thick sections and stained with hematoxylin-eosin for photomicroscopic assessment using a Model N-400ME photomicroscope (CEL-TECH Diagnostics, Hamburg, Germany). The histopathology slides were viewed at various magnifications (×40, ×100, and × 400) to detect pathological lesions.

### Statistical analysis

Data are expressed as mean ± *SD* for body and relative organ weights while data for biochemical and hematological assays are expressed as mean ± s.e.m. Statistical analysis was carried out using One-Way Analysis of Variance (ANOVA) and Newman-Keuls' *post hoc* test. Level of statistical significance was considered at values of *p* < 0.05.

## Results

### Preliminary phytochemical analysis

Tannins, saponins, flavonoids, and presence of a steroidal nucleus (i.e., aglycone portion of cardiac glycoside) were detected in HLE-FP.

### Acute toxicity studies

Single oral treatment of rats with 5000 mg/kg body weight of HLE-FP produced no death within the short- and long-term outcome of the limit dose test of Up-and-Down procedure. Observed behavioral manifestations include dyspnoea, restlessness/agitation, generalized body tremor, feed and water refusal within 24 h post-treatment *p.o*. These manifestations gradually subsided after 24 h. The LD_50_ was estimated to be greater than 5000 mg/kg body weight/oral route.

Single intraperitoneal treatment with HLE-FP at doses of 250, 500, 1000, 2000, and 4000 mg/kg caused 0, 0, 0, 20, and 80% mortality, respectively, in the treated mice within 24 h post-treatment. Using log dose-probit analysis, the *i.p*. LD_50_ was estimated to be 2951.2 mg/kg. Behavioral manifestations sequel to *i.p*. administration of HLE-FP includes restlessness and writhing within the first 15 min, and calmness and anorexia within 48 h post-treatment for surviving mice.

### Effect of HLE-FP on the body weight in the sub-chronic oral toxicity study

Table 1 shows the effect of repeated daily oral treatment with 20, 100, and 500 mg/kg HLE-FP on the average body weight of rats on days 1, 15, and 30, as well as percentage body weight changes on day 30. Oral HLE-FP treatment for 30 days caused significant (*p* < 0.05) reductions in the weight gain pattern of treated rats in a non-dose-dependent manner with the highest reductions recorded for Group III (20 mg/kg) rats while the lowest weight gain reduction was recorded for Group II (100 mg/kg) rats.

**Table 1:** **Effect of HLE-FP on body weight in the sub-chronic oral toxicity study**.

**Group**	**1st day**	**15th day**	**30th day**	**30th day**	**45th day**	**60th day**	**60th day**
	**wt. (g)**	**wt. (g)**	**wt. (g)**	**%Δwt**.	**wt. (g)**	**wt. (g)**	**%Δwt**.
I	120.40 ± 24.25	136.80 ± 17.24	159.10 ± 19.80	34.32 ± 18.47^a^	173.60 ± 18.00	187.40 ± 20.74	55.65 ± 25.06^a^
II	108.80 ± 7.45	134.70 ± 08.09	153.80 ± 21.04	41.67 ± 21.04	164.10 ± 18.19	191.20 ± 16.71	71.92 ± 09.57^a^
III	121.80 ± 17.10	144.00 ± 19.42	161.30 ± 18.71	28.02 ± 32.16^a^	165.90 ± 16.08	191.80 ± 23.51	69.02 ± 29.44^a^
IV	86.33 ± 23.40	129.30 ± 16.31	151.10 ± 12.40	85.62 ± 44.65	163.30 ± 11.05	175.20 ± 18.83	102.94 ± 15.50

a *Represents a significant decrease at p < 0.05 compared to control values (Group IV)*.

*I, 500 mg/kg/day HLE-FP; II, 100 mg/kg/day HLE-FP; III, 20 mg/kg/day HLE-FP; IV, 10 ml/kg normal saline*.

Table 1 also shows the effect of repeated daily oral treatment with 20, 100, and 500 mg/kg of HLE-FP on the average body weight of rats on days 45 and 60, as well as percentage body weight changes of treated rats.

By the 60th day of the study, Group I rats treated with the highest dose of HLE-FP had the least weight gain pattern when compared to untreated control values (Group IV rats) with the most significant weight gain recorded for the Group II rats which were treated with 100 mg/kg/day HLE-FP. This observation suggests that HLE-FP cause significant (*p* < 0.05) non-dose-dependent weight loss effect on the treated rats.

### Effect of HLE-FP on relative organ weight in the sub-chronic oral toxicity study

As shown in Table 2, there was no significant difference (*p* > 0.05) between control (normal saline) values and the HLE-FP treatment groups values in the 30 and 60 days treatment periods. This observation was same in the reversibility study.

**Table 2:** **Effect of HLE-FP on relative organ weight in the sub-chronic oral toxicity study**.

**Group**	**30 days treatment**						**60 days treatment**						**Reversibility study**					
	**Liver**	**Stomach**	**Lungs**	**Spleen**	**Kidneys**	**Heart**	**Liver**	**Stomach**	**Lungs**	**Spleen**	**Kidneys**	**Heart**	**Liver**	**Stomach**	**Lungs**	**Spleen**	**Kidneys**	**Heart**
I	3.40 ± 0.48	1.32 ± 0.23	0.89 ± 0.23	0.66 ± 0.11	0.66 ± 0.11	0.66 ± 0.11	4.18 ± 1.05	1.31 ± 0.29	0.96 ± 0.35	0.59 ± 0.04	0.59± 0.04	0.59 ± 0.04	2.91± 0.24	1.08 ± 0.12	1.08 ± 0.37	0.54± 0.06	0.54 ± 0.06	0.54± 0.06
II	4.01± 0.37	3.80 ± 1.27	0.89 ± 0.40	0.72 ± 0.08	0.72 ± 0.11	0.72 ± 0.11	3.71 ± 0.79	1.31 ± 0.29	1.90 ± 0.92	0.64 ± 0.02	0.64 ± 0.02	0.64 ± 0.02	2.70 ± 0.20	1.05 ± 0.08	1.05 ± 0.08	0.53 ± 0.04	0.53 ± 0.04	0.53 ± 0.04
III	4.21 ± 0.34	3.74 ± 0.50	1.10 ± 0.48	0.84 ± 0.29	0.84 ± 0.29	0.84 ± 0.29	3.66 ± 0.31	1.54 ± 0.28	1.19 ± 0.09	1.19 ± 0.49	1.19 ± 0.49	1.19 ± 0.49	3.26 ± 0.29	1.05 ± 0.32	1.14 ± 0.33	0.53 ± 0.06	0.53 ± 0.06	0.53 ± 0.06
IV	3.49 ± 0.29	1.29 ± 0.03	0.64 ± 0.01	0.65 ± 0.01	0.65 ± 0.01	0.65 ± 0.01	3.85 ± 0.21	1.20 ± 0.04	0.84 ± 0.27	1.21 ± 0.50	1.21 ± 0.50	1.21 ± 0.50	3.54 ± 0.25	1.15 ± 0.13	1.20 ± 0.24	0.58 ± 0.06	0.58 ± 0.06	0.58 ± 0.06

*p > 0.05 compared to control values (Group IV)*.

*I, 500 mg/kg/day HLE-FP; II, 100 mg/kg/day HLE-FP; III, 20 mg/kg/day HLE-FP; IV, 10 ml/kg normal saline*.

### Effect of HLE-FP on serum electrolytes, urea and creatinine levels in the sub-chronic oral toxicity study

Repeated daily oral treatment with 20–500 mg/kg HLE-FP for 30 days caused non-significant and non-dose-dependent (*p* > 0.05) rise in serum Na^+^ concentration (Table 3). 20 and 100 mg/kg HLE-FP also caused significant (*p* < 0.05) increases in serum K^+^ and HCO^−^_3_ concentrations compared to control values, respectively. However, 20–500 mg/kg HLE-FP caused non-significant (*p* > 0.05) alterations in the serum levels of urea and creatinine.

**Table 3:** **Effect of HLE-FP on serum electrolytes, urea and creatinine levels in the sub-chronic oral toxicity study**.

**Group**	**30 days treatment**						**60 days treatment**						**Reversibility study**					
	**Na^+^**	**K^+^**	**Cl^−^**	**HCO^−^_3_**	**Urea**	**Creatinine**	**Na^+^**	**K^+^**	**Cl^−^**	**HCO^−^_3_**	**Urea**	**Creatinine**	**Na^+^**	**K^+^**	**Cl^−^**	**HCO^−^_3_**	**Urea**	**Creatinine**
	**(mmol/L)**	**(mmol/L)**	**(mmol/L)**	**(mmol/L)**	**(mg/dL)**	**(mg/dL)**	**(mmol/L)**	**(mmol/L)**	**(mmol/L)**	**(mmol/L)**	**(mg/dL)**	**(mg/dL)**	**(mmol/L)**	**(mmol/L)**	**(mmol/L)**	**(mmol/L)**	**(mg/dL)**	**(mg/dL)**
I	139.00 ± 1.67	6.60 ± 0.34	105.00 ± 1.18	18.16 ± 0.99	7.96 ± 0.74	43.52 ± 3.14	140.40 ± 0.61^b^	8.12 ± 0.12^a^	104.70 ± 0.48	20.18 ± 0.67	6.38 ± 0.27^a^	60.46 ± 2.09	142.00 ± 1.00	4.34 ± 0.09^b^	103.20 ± 0.86	20.60 ± 0.75	6.66 ± 0.20	57.92 ± 1.88^a^
II	142.60 ± 1.54	7.05 ± 0.25	103.00 ± 1.74	23.60 ± 0.25^a^	6.84 ± 0.70	36.23 ± 5.30	141.20 ± 0.88	7.17 ± 0.25	107.10 ± 1.00	21.46 ± 1.20	7.84 ± 0.16^a^	66.02 ± 4.81	145.60 ± 1.03	5.30 ± 0.26	106.20 ± 0.37	21.00 ± 0.55	7.88 ± 0.14^a^	57.96 ± 1.18^a^
III	140.10 ± 1.54	8.52 ± 0.42^a^	103.60 ± 3.09	18.72 ± 1.78	8.12 ± 1.16	31.30 ± 2.37	144.70 ± 1.04	6.43 ± 0.34	106.00 ± 0.93	23.26 ± 0.70^a^	8.54 ± 0.12^a^	67.69 ± 1.92	143.20 ± 1.02	4.94 ± 0.34^b^	103.20 ± 0.86	20.00 ± 0.63	8.78 ± 0.31^a^	52.00 ± 1.47^a^
IV	136.50 ± 1.92	6.74 ± 0.42	104.40 ± 0.68	19.06 ± 0.83	6.24 ± 0.54	38.30 ± 5.79	144.60 ± 1.46	6.17 ± 0.33	104.30 ± 0.40	19.16 ± 0.77	5.32 ± 0.09	60.06 ± 1.81	144.00 ± 1.84	6.32 ± 0.44	104.40 ± 0.81	17.80 ± 1.69	6.26 ± 0.16	45.30 ± 1.98

a and b *represent a significant increase and decrease at p < 0.05 respectively, compared to control values (Group IV)*.

*I, 500 mg/kg/day HLE-FP; II, 100 mg/kg/day HLE-FP; III, 20 mg/kg/day HLE-FP; IV, 10 ml/kg normal saline*.

Repeated oral treatments with 20–500 mg/kg HLE-FP for 60 days was associated with dose-related reductions in the serum levels of Na^+^ with the most significant (*p* < 0.05) reduction recorded at 500 mg/kg of HLE-FP while also causing dose-dependent increase in the serum levels of K^+^ with the most significant (*p* < 0.05) increase also recorded at 500 mg/kg HLE-FP compared to control values (Table 3). Oral treatment with 20 mg/kg HLE-FP caused significant (*p* < 0.05) increase in the serum level of HCO^−^_3_ while 20–500 mg/kg HLE-FP caused significant (*p* < 0.05) increase in the serum levels of urea compared to control values. However, oral treatment with 20–500 mg/kg HLE-FP for 60 days was associated with non-significant (*p* > 0.05) alterations in the serum Cl^−^ and creatinine levels.

Withdrawal of repeated daily oral treatment with 20–500 mg/kg HLE-FP for 15 days caused reductions in the serum K^+^ concentration, significantly (*p* < 0.05) at doses of 500 and 20 mg/kg compared to the control value (Table 3). However, withdrawal of HLE-FP from the treated rats for 15 days was associated with significant (*p* < 0.05) and persistent increase in serum urea concentrations at 20 and 100 mg/kg when compared to the control value. Also, there was significant (*p* < 0.05) increase in the serum creatinine concentrations in all the treatment groups compared to the control value. On Na^+^, Cl^−^ and HCO^−^_3_ concentrations, withdrawal of HLE-FP caused non-significant alterations in serum levels compared to control values.

### Effect of HLE-FP on other serum biochemical parameters in the sub-chronic oral toxicity study

Repeated oral treatment with 500 mg/kg HLE-FP for 30 days was associated with significant (*p* < 0.05) increases in the serum total protein, triglyceride and high density cholesterol levels when compared to control values (Table 4). HLE-FP at 100 mg/kg also caused significant (*p* < 0.05) increase in serum triglyceride and decrease in serum total protein compared to control values. However, HLE-FP at 20–500 mg/kg caused non-significant (*p* > 0.05) alterations in the serum levels of ALT, AST, ALP, albumin and total cholesterol compared to control values.

**Table 4:** **Effect of HLE-FP on other serum biochemical parameters in the sub-chronic oral toxicity study**.

**Group**	**I**	**II**	**III**	**IV**
**30 DAYS TREATMENT**				
ALT (U/L)	102.60 ± 22.92	72.15 ± 15.71	104.30 ± 18.15	77.19 ± 13.28
AST (U/L)	573.40 ± 89.40	440.80 ± 61.18	508.90 ± 49.72	507.20 ± 75.87
ALP (U/L)	113.70 ± 33.20	145.60 ± 18.45	151.70 ± 12.38	139.10 ± 26.10
TP (mg/L)	78.64 ± 1.64^a^	55.84 ± 3.38^b^	63.22 ± 3.10	68.66 ± 2.71
ALB (mg/L)	41.60 ± 1.18	30.62 ± 2.58	34.32 ± 1.71	36.14 ± 1.61
TG (mg/L)	1.13 ± 0.41^a^	1.13 ± 0.13^a^	0.81 ± 0.12	0.66 ± 0.03
TC (mg/L)	2.30 ± 0.17	1.84 ± 0.14	1.77 ± 0.23	1.94 ± 0.11
HDL-c (mg/L)	31.20 ± 4.27^a^	17.00 ± 3.11	22.20 ± 2.75	12.80 ± 2.44
**60 DAYS TREATMENT**				
ALT (U/L)	70.56 ± 22.92	78.90 ± 15.64	128.40 ± 1.00	91.96 ± 5.46
AST (U/L)	487.90 ± 53.02	466.50 ± 28.71	691.10 ± 25.48	534.90 ± 39.48
ALP (U/L)	155.70 ± 17.46	219.70 ± 70.48	192.50 ± 11.14	120.50 ± 2.41
TP (mg/L)	71.72 ± 2.95^a^	77.46 ± 1.37^a^	67.08 ± 1.78	67.24 ± 1.21
ALB (mg/L)	40.24 ± 2.07	38.26 ± 1.19	33.66 ± 0.30	37.62 ± 0.49
TG (mg/L)	1.11 ± 0.08^a^	0.75 ± 0.03	0.88 ± 0.08	0.72 ± 0.04
TC (mg/L)	1.55 ± 0.28	0.75 ± 0.03	0.88 ± 0.08	0.72 ± 0.04
HDL-c (mg/L)	19.40 ± 0.75	19.40 ± 0.81	21.60 ± 0.87	19.20 ± 0.58
**REVERSIBILITY STUDY**				
ALT (U/L)	88.57 ± 7.81^b^	67.97 ± 4.37^b^	90.98 ± 3.91	92.62 ± 1.57
AST (U/L)	558.00 ± 23.23	630.30 ± 19.15	575.70 ± 16.54	558.90 ± 27.60
ALP (U/L)	166.40 ± 2.47	216.20 ± 26.38^a^	186.70 ± 7.24	147.70 ± 14.26
TP (mg/L)	66.56 ± 1.13	69.96 ± 0.77	70.96 ± 2.26	70.04 ± 1.19
ALB (mg/L)	36.58 ± 1.28	39.18 ± 0.61	38.78 ± 1.04	37.02 ± 0.41
TG (mg/L)	0.98 ± 0.06	0.85 ± 0.03	0.99 ± 0.07	0.92 ± 0.03
TC (mg/L)	2.27 ± 0.20	1.77 ± 0.05	1.85 ± 0.07	2.10 ± 0.18
HDL-c (mg/L)	19.80 ± 1.46	25.80 ± 2.42	22.40 ± 1.12	22.60 ± 2.14

a and b *represent significant increase and decrease at p < 0.05 compared to control values, respectively*.

*I, 500 mg/kg/day HLE-FP; II, 100 mg/kg/day HLE-FP; III, 20 mg/kg/day HLE-FP; IV, 10 ml/kg normal saline*.

Repeated oral treatment at 20 mg/kg of HLE-FP for 60 days was associated with non-significant (*p* > 0.05) increase in the serum ALT and AST levels, while there was also a non-significant (*p* > 0.05) decrease in the serum ALT and AST levels at 100 and 500 mg/kg HLE-FP compared to control values (Table 4). 500 mg/kg HLE-FP also caused significant (*p* < 0.05) increases in the serum levels of triglyceride and total cholesterol compared to control values. HLE-FP at doses of 100 and 500 mg/kg also significantly (*p* < 0.05) elevated total protein serum level compared to control values. However, oral treatment with 20–500 mg/kg HLE-FP for 60 days caused non-significant (*p* > 0.05) alterations in the serum albumin and high density cholesterol levels compared to control values. In respect of ALP, non-significant increases were observed at all HLE-FP doses relative to control.

Withdrawal of oral treatment with HLE-FP was associated with significant (*p* < 0.05) reduction in the serum levels of ALT at doses of 100 and 500 mg/kg HLE-FP compared to control values (Table 4). Also, withdrawal of oral treatment with HLE-FP for 15 days was associated with non-significant (*p* > 0.05) alterations in the serum levels of AST, total protein, albumin, triglyceride, total cholesterol and high density cholesterol.

### Effect of HLE-FP on hematological parameters in the sub-chronic oral toxicity study

Repeated oral treatment with HLE-FP for 30 days was associated with significant (*p* < 0.05) increase in Hb, PCV and RBC at 500 mg/kg compared to control values (Table 5).

**Table 5:** **Effect of HLE-FP on hematological parameters in the sub-chronic oral toxicity study**.

**Group**	**I**	**II**	**III**	**IV**
**30 DAYS TREATMENT**				
Hb (g/dL)	13.28 ± 0.15^a^	11.24 ± 0.22	10.76 ± 0.64	11.26 ± 0.29
PCV (%)	48.58 ± 1.03^a^	42.96 ± 1.47	38.78 ± 2.01	41.66 ± 0.72
RBC (×10^6^/L)	8.13 ± 0.22^a^	7.03 ± 0.27	6.38 ± 0.31	6.69 ± 0.08
MCV (fL)	59.86 ± 1.08	61.12 ± 0.83	60.80 ± 1.23	62.30 ± 1.29
MCH (pg)	16.36 ± 0.40	16.60 ± 0.77	17.04 ± 0.91	16.84 ± 0.39
PLT (×10^3^/L)	637.80 ± 200.10	584.20 ± 54.64	648.20 ± 34.89	667.80 ± 41.56
WBC (×10^3^/L)	10.72 ± 1.56	14.12 ± 2.59	12.62 ± 2.31	17.36 ± 1.95
%LYM	72.92 ± 3.03	85.88 ± 0.96	83.76 ± 4.14	79.18 ± 6.30
%NEU	16.34 ± 2.38	8.46 ± 0.71	10.18 ± 3.15	11.32 ± 3.21
%MXD	10.74 ± 0.90	5.66 ± 0.60	6.06 ± 1.13	9.50 ± 3.18
**60 DAYS TREATMENT**				
Hb (g/dL)	12.68 ± 0.33	13.06 ± 0.38	11.66 ± 0.78	12.08 ± 0.29
PCV (%)	45.80 ± 1.23	44.88 ± 1.17	43.96 ± 1.48	44.08 ± 1.29
RBC (×10^6^/L)	7.82 ± 0.12	7.73 ± 0.15	7.95 ± 0.21	8.01 ± 0.37
MCV (fL)	58.50 ± 0.74^a^	58.02 ± 0.89^a^	55.32 ± 0.79	54.28 ± 0.69
MCH (pg)	16.20 ± 0.26^a^	16.90 ± 0.35^a^	15.24 ± 0.29	15.18 ± 0.40
PLT (×10^3^/L)	621.20 ± 44.94	706.80 ± 73.92	696.40 ± 97.85	473.40 ± 38.06
WBC (×10^3^/L)	14.18 ± 1.29	12.24 ± 0.86	14.60 ± 1.15	14.02 ± 2.03
%LYM	83.06 ± 1.78	77.56 ± 3.35	75.02 ± 3.95	81.42 ± 2.23
%NEU	10.34 ± 1.78	14.76 ± 2.85	18.26 ± 4.28	12.50 ± 2.44
%MXD	6.60 ± 0.48	7.68 ± 1.04	6.72 ± 1.07	6.08 ± 0.82
**REVERSIBILITY STUDY**				
Hb (g/dL)	12.18 ± 0.17	12.26 ± 0.31	12.48 ± 0.93	12.62 ± 0.42
PCV (%)	47.88 ± 3.80	50.14 ± 1.41	50.46 ± 1.18	49.12 ± 0.91
RBC (×10^6^/L)	7.22 ± 0.53	7.76 ± 0.19	11.13 ± 1.75	7.90 ± 0.12
MCV (fL)	66.26 ± 1.38	64.66 ± 1.19	61.36 ± 0.97	62.22 ± 0.17
MCH (pg)	17.34 ± 1.65	15.82 ± 0.30	15.20 ± 0.18	15.96 ± 0.27
PLT (×10^3^/L)	710.20 ± 29.53	606.40 ± 66.64	710.00 ± 187.10	726.40 ± 30.86
WBC (×10^3^/L)	14.38 ± 3.23	9.72 ± 0.47	12.20 ± 1.58	16.08 ± 1.76
%LYM	85.18 ± 1.46	85.14 ± 2.37	85.00 ± 2.59	77.76 ± 0.96
%NEU	4.94 ± 0.32	5.76 ± 0.83	3.71 ± 0.67^b^	6.25 ± 0.09
%MXD	9.88 ± 1.65	9.10 ± 1.60	11.48 ± 1.87	16.02 ± 1.03

a and b *represent significant increase and decrease at p < 0.05 compared to control values, respectively*.

*I, 500 mg/kg/day HLE-FP; II, 100 mg/kg/day HLE-FP; III, 20 mg/kg/day HLE-FP; IV, 10 ml/kg normal saline*.

By the 60th day of oral treatment with 20–500 mg/kg HLE-FP, there were significant (*p* < 0.05) increases in MCV and MCH indices at 100 and 500 mg/kg compared to control values (Table 5).

Withdrawal of oral treatment with HLE-FP for 15 days resulted in the reversal of earlier elevated hematological indices on 30th (Hb, PCV and RBC) and 60th (MCV and MCH) days with values comparable and not significantly different (*p* > 0.05) from control values (Table 5). All other measured hematological parameters were not significantly (*p* > 0.05) different from control except for %NEU in respect of which a significant reduction (*p* < 0.05) was observed at the dose of 20 mg/kg compared to the control value.

### Histopathological assessment

**Figure 1:**
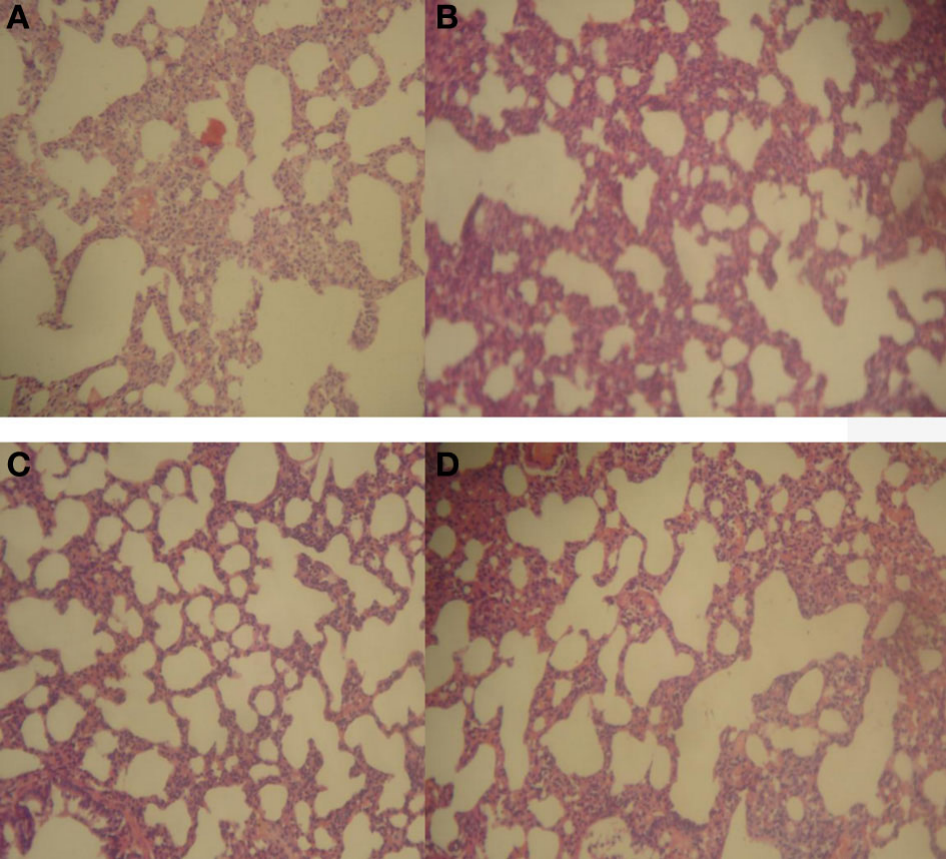
**Representative sections of rat lungs in respect of 30 days treatment**. **(A)** Normal rat lung showing normal alveoli and lung parenchyma; **(B)** 20 mg/kg/day HLE-FP-treated lung showing hyperplasia of the alveolar septae resulting in thickened alveolar space; **(C)** 100 mg/kg/day HLE-FP-treated lung showing alveolar space smaller than that of control; and **(D)** 500 mg/kg/day HLE-FP-treated lung showing alveolar space remarkably smaller than that of control (×100 magnification).

Repeated oral treatment with 20–500 mg/kg HLE-FP for 30 days was associated with increasing alveolar septa thickness and reduction in alveolar space compared to control lungs (Figure 1). However, no significant histological lesions were observed in respect of the liver, stomach, kidneys, spleen and heart.

**Figure 2:**
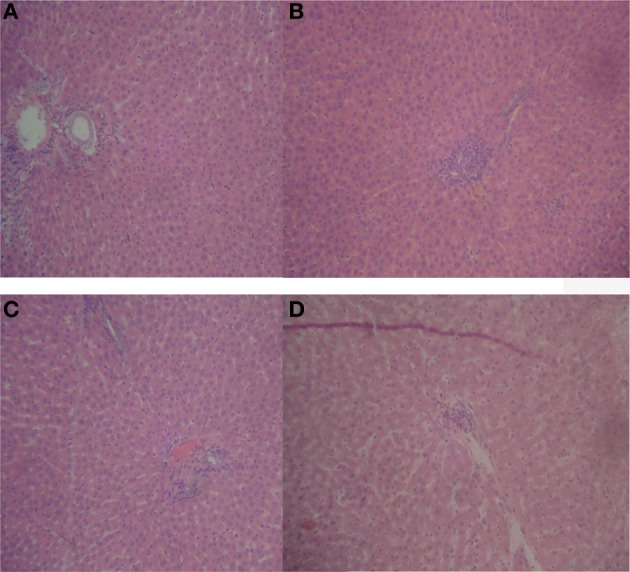
**Representative sections of rat liver in respect of 60 days treatment**. **(A)** Normal rat liver showing normal hepatic architecture; **(B)** 20 mg/kg/day HLE-FP-treated rat liver showing patchy areas of lymphocytic infiltrations; **(C)** 100 mg/kg/day HLE-FP-treated rat liver showing mild vascular congestion; and **(D)** 500 mg/kg/day HLE-FP-treated rat liver showing scattered areas of lymphocytic aggregates with mild sinusoidal congestion signifying mild-to-moderate inflammation (× 100 magnification).

**Figure 3:**
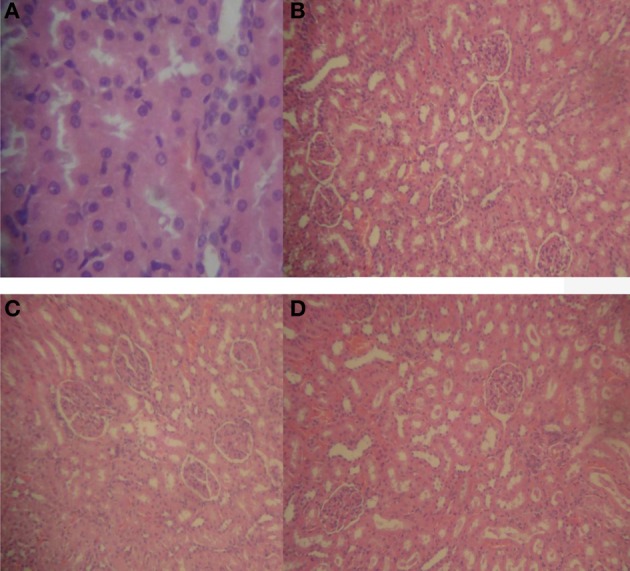
**Representative sections of rat kidneys in respect of 60 days treatment**. **(A)** Normal rat kidney showing regular tubular cells with normal nuclei; **(B)** 20 mg/kg/day HLE-FP-treated rat kidney showing glomeruli with capillary vascular congestion; **(C)** 100 mg/kg/day HLE-FP-treated rat kidney showing regular glomeruli with scattered localized hemorrhage; and **(D)** 500 mg/kg/day HLE-FP-treated rat kidney showing regular glomeruli and tubules with moderate vascular congestion (× 100, × 400 magnification).

**Figure 4:**
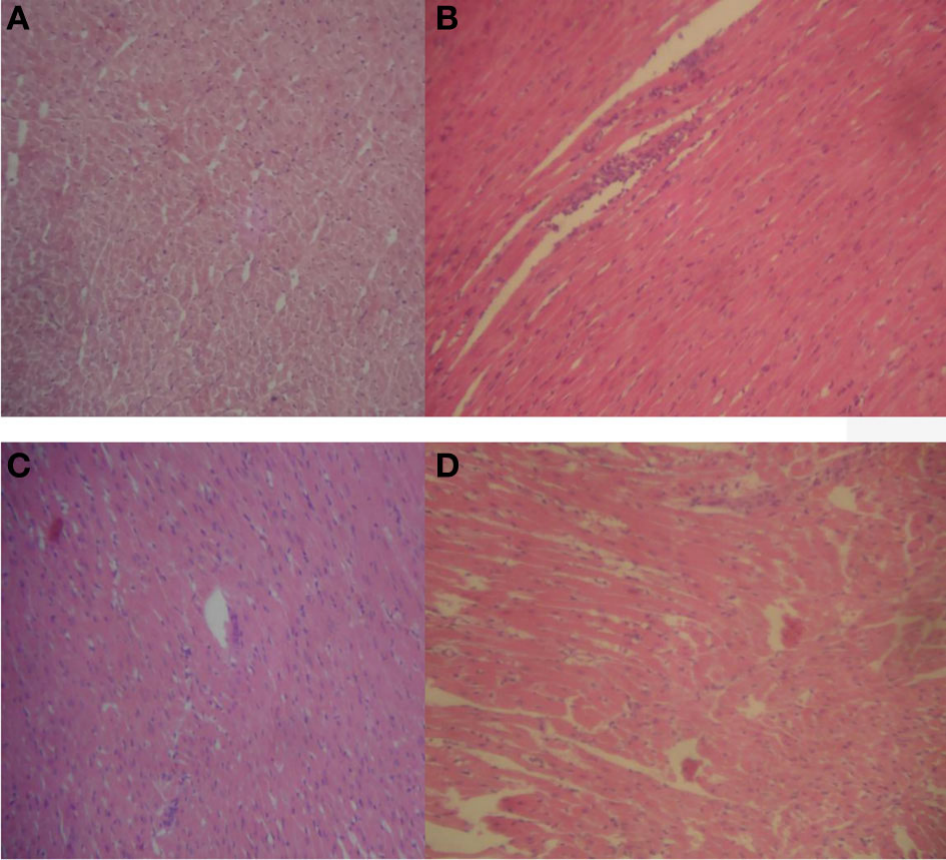
**Representative sections of rat heart in respect of 60 days treatment**. **(A)** Normal rat heart showing normal cardiac myocytes and fascicules; **(B)** 20 mg/kg/day HLE-FP-treated rat heart showing few lymphocytic infiltrations around the cardiac myocytes and fascicules; **(C)** 100 mg/kg/day HLE-FP-treated rat heart showing no remarkable histological lesions; and **(D)** 500 mg/kg/day HLE-FP-treated rat heart showing occasional lymphocytic aggregates around the cardiac myocytes (× 100 magnification).

With prolonged oral treatment with 20–500 mg/kg HLE-FP for 60 days, there were scattered areas of lymphocytic aggregates with varying degrees of sinusoidal congestion signifying mild-to-moderate inflammatory process in the liver compared to the control liver (Figure 2). The kidneys also showed glomeruli with capillary vascular congestion and scattered areas of haemorrhage compared to the control kidney (Figure 3). In respect of the heart, few lymphocytic infiltrations around the cardiac myocytes and fascicules were observed in the 20 mg/kg/day HLE-FP-treated group while occasional lymphocytic aggregates were observed around the cardiac myocytes in the 500 mg/kg/day HLE-FP-treated group compared to control (Figure 4). There were no remarkable histopathological changes observed in the lungs, stomach, and spleen.

### Effect of 15 days toxicity reversibility of oral treatment with 20–500 mg/kg/day of HLE-FP on histopathology of selected vital organs of treated rats

**Figure 5:**
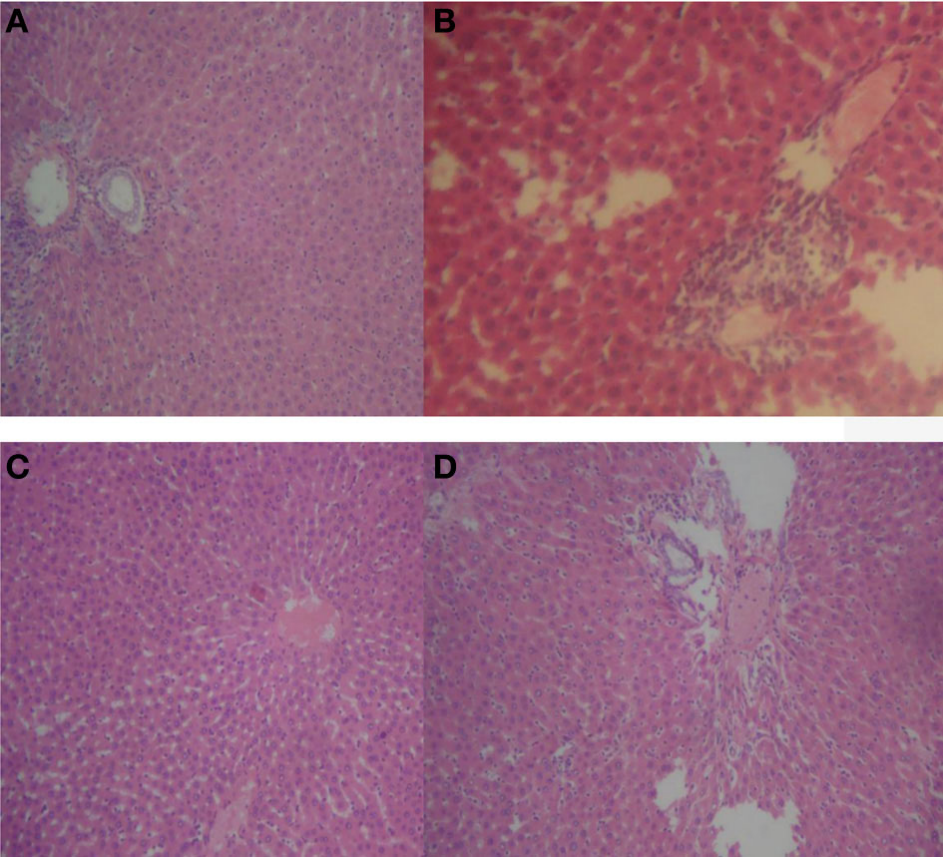
**Representative sections of rat liver in respect of reversibility study**. **(A)** Normal rat liver showing normal hepatocytes and hepatic vasculature; **(B)** 20 mg/kg/day HLE-FP-treated rat liver showing epithelial proliferation and scattered areas of vascular congestion; **(C)** 100 mg/kg/day HLE-FP-treated rat liver showing scattered areas of vascular congestion; and **(D)** 500 mg/kg/day HLE-FP-treated rat liver showing few local lymphocytic aggregates with mild-to-moderate vascular congestion (× 100 magnification).

**Figure 6:**
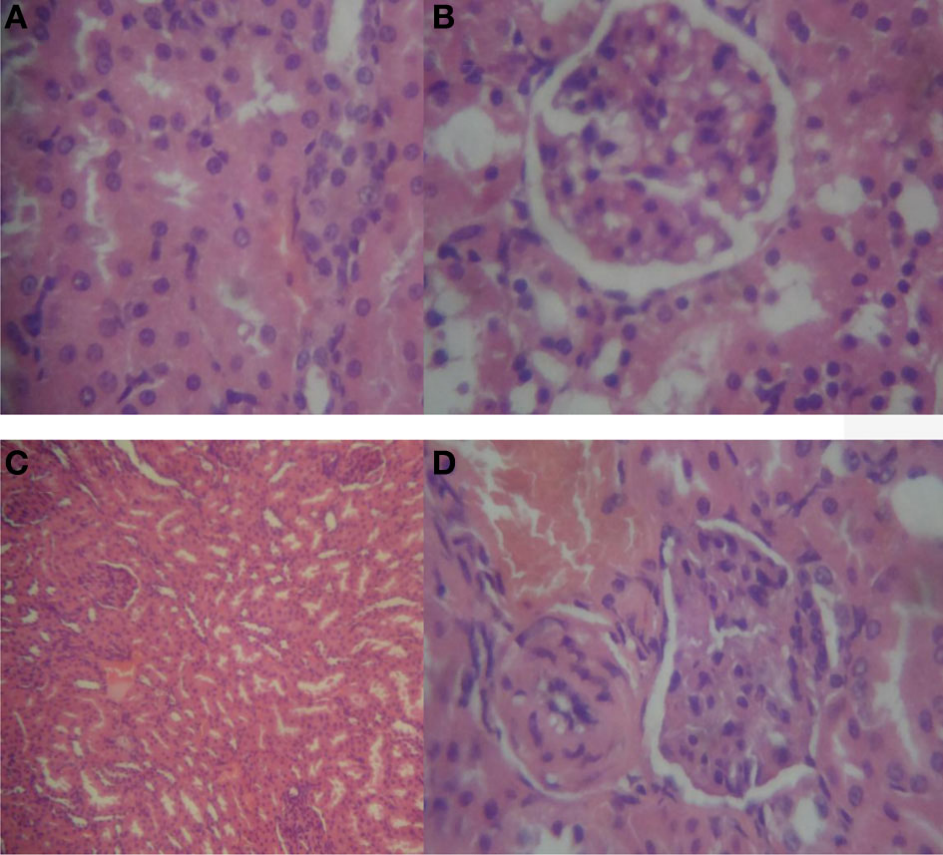
**Representative sections of rat kidneys in respect of reversibility study**. **(A)** Normal rat kidney showing regular tubular cells with normal nuclei; **(B)** 20 mg/kg/day HLE-FP-treated rat kidney showing focal area of epithelial cell proliferation; **(C)** 100 mg/kg/day HLE-FP-treated rat kidney showing congested vascular channels and areas of hemorrhage; and **(D)** 500 mg/kg/day HLE-FP-treated rat kidney showing localized hemorrhage and glomerular capillary congestion (×100, × 400 magnification).

No remarkable histological changes were observed in the lungs, stomach, spleen, and heart. However, persistent few focal lymphocytic infiltrations with varying mild-to-moderate vascular congestion were observed in the liver (Figure 5) while varying degrees of glomerular congestions were observed in the kidneys (Figure 6).

## Discussion

Toxicity studies have always been considered a vital integral component of drug development bearing in mind that herbal medicines are often used indiscriminately without recourse to the potential side-effects which could vary from being mild, moderate, and severe, to life-threatening (World Health Organization, 1987, 2000). In the present study, the acute and sub-chronic toxicity, and toxicity reversibility profile of the hydroethanolic leaf extract of *F. paniculata* cav. (HLE-FP) were evaluated in rodents using anthropometric, biochemical, hematological and histopathological parameters.

The results of acute oral and intraperitoneal toxicity studies showed the LD_50_ values of HLE-FP to be greater than 5000 mg/kg and 2951.2 mg/kg, respectively. The limit test is primarily used in situations where the investigator has information indicating that the test material is likely to be non-toxic or of low toxicity (OECD, 2002a,b) considering the fact that studies have shown that extracts with acute oral LD_50_ value of greater than 3000–5000 mg/kg can be considered relatively safe (Chan et al., 1982; Itthipanichpong et al., 1987; OECD, 2008a). Thus, the high LD_50_ values obtained for HLE-FP in this study are strong pointers to the fact that HLE-FP could be considered safe on acute exposure to the extract.

Sub-chronic oral treatment with 20–500 mg/kg/day of HLE-FP over the period of 60 days was associated with significant decrease in the weight gain pattern of HLE-FP-treated rats in a non-dose dependent manner. This result suggests that HLE-FP may be having weight losing effect mediated through either appetite-inhibiting or nutrient losing mechanisms. Unfortunately, in this present study, the food consumption pattern of HLE-FP-treated rats was not investigated and this would have provided insight into the possible weight losing potential of HLE-FP. However, literature has shown that saponins cause weight loss through appetite inhibition and inhibition of nutrients absorption from the intestinal lumen (Ajagbonna et al., 1999). Thus, the presence of saponins alone or in combination with other weight-losing phytoconstituents in HLE-FP could account for the weight losing effect of HLE-FP.

On repeated daily exposure of rats to HLE-FP for 30 days, there was profound increment in hematological parameters such as hemoglobin, hematocrit and red blood cell counts without significant alterations to all other measured hematological parameters at the dose of 500 mg/kg HLE-FP. By the 60th day of repeated oral treatment, there was increment in the mean corpuscular volume and mean corpuscular hemoglobin still at the dose of 500 mg/kg HLE-FP. However, these increments in the hematological parameters were reversed to about normal after oral treatment with HLE-FP was stopped. This observation strongly suggests that HLE-FP could have stimulatory effect on the pluripotent red cell lines in the bone marrow. Further studies will still be required to validate this hypothesis. The hematopoietic system is one of the most sensitive targets of toxic compounds and it is thus considered an important index of physiological and pathological status in man and animals (Feldman et al., 2000; Adeneye et al., 2006). Also, hematological parameters provide vital information regarding the status of bone marrow activity and intravascular effects such as hemolysis and anemia (Voigt, 2000).

On the liver function, sub-chronic effects of 20–500 mg/kg/day of HLE-FP were also evaluated. Oral treatment with 500 mg/kg/day HLE-FP for 30 days was observed to have resulted in profound elevation in the serum levels of total protein, triglyceride, and high density cholesterol and total cholesterol which were sustained over additional 30 days. However, there were no significant alterations in serum liver enzymes (ALT, AST, and ALP) values and other hepatic function parameters such as total protein and albumin, indicating that HLE-FP did not cause deterioration in the liver function. Although these biochemical results appear to be at variance with that of its histopathology which showed scattered areas of lymphocytic aggregates with mild sinusoidal congestion, it could mean that the histological lesions were not severe enough so as to have reflected in the measured hepatic enzyme markers and other hepatic function tests. The liver is known to be the main organ of detoxification for most drugs and alterations in its enzyme markers are considered strong indicators of toxicity profile of a drug (Woodman, 1996). Thus, the fact that HLE-FP caused no significant alterations in these hepatic enzyme markers suggest that HLE-FP is not hepatotoxic since hepatotoxicity is marked by profound elevations in the serum levels of ALT, AST, ALP and at times reduced serum total protein and albumin levels (Caisey and King, 1980; Woodman, 1996).

On the renal function integrity, repeated daily oral treatment with 20–500 mg/kg/day HLE-FP for 30 days was associated with significant elevation in the serum potassium and bicarbonate levels with no alterations in other parameters such sodium, chloride, urea and creatinine. With further oral treatment for additional 30 days, there were profound elevations in the serum potassium, bicarbonates, and urea levels suggesting deleterious effect on the renal function. The compromise in the renal function was corroborated by the histopathological findings which showed areas of localized hemorrhage and glomerular capillary congestion. Renal functions are measured by serum electrolytes, urea and creatinine, thus, elevations in the serum levels of these parameters are indicative of renal injury or insult (Caisey and King, 1980; Woodman, 1996; Levine, 2002). These changes were irreversible with the extract withdrawal for 15 days.

## Conclusion

The results obtained in this study suggest that although HLE-FP may be non-toxic on acute exposure and generally safe on sub-chronic administration, this herbal preparation may have deleterious effects on renal functions of rats treated with it over a prolonged period of time with these toxicities being irreversible with withdrawal of the extract for 15 days. These observations suggest that decoction made from the leaf of *F. paniculata* should be used with caution in order to avoid renal toxicities.

### Conflict of interest statement

The authors declare that the research was conducted in the absence of any commercial or financial relationships that could be construed as a potential conflict of interest.
